# Language of mothers and fathers in interaction with their autistic children

**DOI:** 10.3389/fpsyt.2023.1254563

**Published:** 2023-11-28

**Authors:** Silvia Perzolli, Arianna Bentenuto, Simona de Falco, Paola Venuti

**Affiliations:** Laboratory of Observation, Diagnosis, and Education (ODFLab), Department of Psychology and Cognitive Science, University of Trento, Rovereto, Italy

**Keywords:** autism, mother-child, father-child, parental speech, interaction

## Abstract

**Introduction:**

Verbal language is one of the most immediate and significant means parents have to express affect and information to their children. Parental speech directed to children has been thoroughly examined in typical development. However, the characteristics of parental speech directed to children with neurodevelopmental disorders are far less well documented, and no recent studies have been carried out that involve autistic^1^ children and their fathers. Therefore, the present study aims to analyze and compare maternal and paternal speech directed to young autistic children, focusing on fathers’ elements of speech in comparison with maternal language.

**Methods:**

*N* = 88 dyads participated in this study. 44 autistic children (41 males and 3 females) (chronological age: *M* = 40.01 months; SD = 11.96) in interaction with their fathers (paternal age *M* = 41.84 years; SD = 7.02) and the same 44 children in interaction with their mothers (maternal age *M* = 37.37 years; SD = 5.45). The language was verbatim transcribed using ELAN software (ELAN Version 6.4, 2022) and coded with an observational tool (Penman) for analyzing functions and referents of speech after reaching a satisfactory level of agreement between two independent transcribers.

**Results:**

No differences emerged considering the affective aspects of speech. However, mothers seem to direct more informative salient statements (*W* = 1,259; *p* = 0.02) and call the child’s attention more often than fathers (*W* = 1,253.5; *p* = 0.02). Regarding referents of informative speech, fathers focused more on the child’s internal states rather than mothers (*W* = 727; *p* = 0.04).

**Discussion:**

These results reveal that fathers seem to display a relationship-based approach focused on a non-intrusive style with few demands while talking with their children, providing a complementary role to mothers that allows complete and harmonious stimulation of all areas of child development.

## 1 Introduction

### 1.1 Parental language and communication

Parent–child communication is characterized by a bidirectional nature: parental language influences child linguistic development, which in turn affects paternal and maternal speech characteristics (1, 2). Communication can be considered the *engine* of each social relationship and involves different aspects such as availability, listening and mutual understanding. In the context of parenting, communication represents a fundamental tool to express needs, affect, and emotions in various ways, including language, smiles, laughs, eye contact, and others. In fact, no aspect of the interaction between parent and child does not involve communication (1). Furthermore, the literature suggests that more responsive communication as well as a wider variety of inputs displayed by parents, result in children’s more complex language use (3, 4).

Parents tend to modulate their speech based on their child competencies and the parental representations they have of their child developmental level. On the other hand, the parents’ contingent and adequate communication supports the child harmonious development. Through language, parents provide salient elements that may reflect more general parenting purposes of scaffolding the child’s cognitive development within an affective framework (5). In general, the literature on typical development focuses on the associations between parental responsiveness in social interactions and subsequent child language ability (3, 4, 6). In addition, children seem to benefit more from both caregivers who display complementary styles of communication, leading to more harmonious development (7–9), suggesting the importance of considering both parents in the investigation of parental linguistic behaviors.

The parental ability to appropriately respond to the child’s verbal and non-verbal signals is called *responsiveness,* and parents that tend to adapt their language according to their child abilities are defined as *verbally responsive* and represent an important element in developing the child’s language, communication, and cognitive functioning (10).

On the contrary, when parents use intrusive statements and commands without considering the child’s abilities or interests, perhaps interfering with child activities, they adopt what’s called a *directive style*.

Parental language can be categorized considering both its style and specific purpose. Different approaches have been used to study parent speech directed to their children, and one of the most relevant concerns the functional aspects of language. Usually, the classification of functional analysis distinguishes between, on the one hand, a linguistic approach that aims to convey information and, on the other hand, a linguistic modality that expresses social, affective content up to emotions (5, 11–13). When language conveys information about children’s experiences, we refer to an *information-salient speech* (e.g., propositional phrases including questions, direct statements, and reports about the child and the environment) (13–16). Instead, when language is mainly used to express affection and to maintain and regulate social exchanges, we refer to an *affective-salient speech* (e.g., encouragements, laughs, songs, onomatopoeias).

Parents may vary in the proportional use of these two different categories. Moreover, with respect to parents may vary in the proportion *information-salient speech* some parents may show an interactive style that engages in reciprocal verbal exchanges, asking children more questions and avoiding direct statements. On the contrary, other parents may tend to use a style characterized by more direct statements and fewer questions.

### 1.2 Parental language and communication in the context of autism

Although recent literature supports the idea that child development is stimulated and supported mainly by the parental responsive style, some research revealed that parents of children in clinical conditions, especially in families with preschoolers with neurodevelopmental disorders, tend to use a more directive style [for a review see (17)]. Autism constitutes one of the most prevalent neurodevelopmental disorders, with a prevalence of 1:36 individuals in the USA (18) and 1:77 in Italy (19). This condition is characterized by social-communicative impairment and restrictive and repetitive behavior patterns (20). Given the socio-communicative and language difficulties of autistic children (20), the structure and linguistic inputs that parents provide for these children appear to be even more essential than for children with typical development (21–23). In the context of autism, more research focused on child factors (e.g., cognitive functioning and symptoms severity) and subsequent children’s linguistic outcomes (24–27). However, less investigation focused on understanding the parents’ linguistic style while interacting with their autistic children and accounting for differences between mothers and fathers that might be relevant in the implementation of adequate activities with their autistic children to optimize children’s learning.

Some research highlighted in a sample of 50 preschoolers with a chronological age of 39 months and a mental age of 13.8 months (23) found an association between parent speech (e.g., mean length utterance, responsiveness) and verbal communication of autistic children. Moreover, in a study by Choi and colleagues (28), 87 dyads were analyzed; 53 children were at high risk of developing autism, and 33 children were at low risk. Results revealed that the mean length of utterance of parents at 18 months was associated with child abilities at 24 months, regardless of the risk of developing autism. Furthermore, investigators analyzing the role of parents’ linguistic features on autistic children’s developmental outcomes reported that parents’ use of utterances with longer phrases (29–31) and more noun types (32) are associated with children’s higher word production. Also, when parents produce a wide variety of questions, children are generally more able to produce and understand complex sentences (33). Until now, most research on parental speech directed to autistic children has focused on morphological characteristics their association with child linguistic outcomes. However, to our knowledge, the functional characteristics of parental speech have been far less investigated, although they might be relevant for understanding parent–child interaction and for implementing early interventions with parental involvement in the context of autism. The few existing studies investigated maternal language (34, 35) and almost ignored fathers’ speech, although father-child healthy relationships have been proven to promote better child developmental outcomes (36). Specifically, these studies reported that maternal language directed to autistic children, compared to that directed to typically developing children, presented enhanced attempts of calling the child’s name, more directive sentences, and fewer questions, as well as more references to child’s actions and the mother herself, together with fewer references to the environment (11, 37). Furthermore, in a recent study that compared three different samples of mother–child dyads of children with a mental age of 24.6 months, results revealed that mothers of autistic children provided a more significant amount of partial repetition compared to mothers of children with typical development and Down Syndrome, probably compensating for their children’s lack of communicative responses (11).

A recent study compared fathers of autistic children to fathers of children with typical development, highlighting a supportive and non-intrusive style of fathers directed to their children with a chronological age of 42.5 and a mental age of 29.4 (38). Fathers of autistic children did not display a more intrusive approach compared to fathers of typical developing children, showing different patterns with respect to the more intrusive behaviors of mothers of autistic children compared to mothers of typical developing children or children with Down Syndrome (11).

When linguistic aspects of mothers and fathers are compared, studies are often inconclusive and few studies investigated functional speech similarities and/or differences of parents of autistic children. In a recent sibling study (39) no differences in the total amount of descriptions of the child’s internal states e that parents directed to their toddlers at high and low risk for ASD were reported. Fathers of infants (0–18 months) at risk for developing autism seem to direct more speech to their infants than fathers of infants with typical development (TD), which stimulates more responses and an increase in the participated behavior of the children (40). Other authors (5) analyzed the differences between responsiveness verbal behaviors used by mothers and fathers and linguistic skills of 16 autistic children with a chronological age range between 40 and 69 months (6). Results revealed that fathers produced fewer statements that referenced or were semantically associated to their child’s attentional focus than mothers. In a subsequent study, Flippin (41) found that father-child speech during the interactions tended to be more direct than mother–child language, however, these findings rely on a single case study. To our knowledge, the only study that compares fathers (and mothers) of autistic children to fathers (and mothers) of children with TD reported substantial similarities between parents of the two groups of children (42).

Taken together, previous findings suggest that mothers of autistic children, when compared to mothers in different conditions, display a more controlling and directive style, intending to convey information (informative-salient speech). In contrast, fathers of autistic children when compared to fathers in different conditions, seem to show a style focused on descriptions and verbalizations of the child’s internal states (e.g., while the child is smiling, the fathers define the child’s emotional state) that may facilitate dyadic interaction.

When fathers’ language is compared to maternal one, part of the literature suggests that fathers make fewer statements with a lower proportion of verbal responses (5, 43), but results are still inconclusive. Although empirical efforts have been conducted so far in understanding the linguistic aspects of parental interactions, i.e., grammatical structure, mean length utterance, and number of words, new research should focus on the functional aspects of the speech delivered by parents as an additional important element to characterize better the way that parents use to talk with their children (44–46).

In line with this, to investigate the functional aspects of speech of mothers and fathers in interaction with their preschool autistic children, we delineated three main research questions with specific hypotheses.

1. Are there any similarities and differences in parental functional speech directed to preschool autistic children?

Specifically, we defined the following hypotheses:

- We expect mothers to display more *informative-salient speech* than fathers. Specifically, in line with previous findings, we expected mothers to call the child’s name more frequently than fathers (10, 11).

Concerning *affective-salient speech*, we hypothesized to find similarities between the two caregivers in light of recent findings reporting similar levels of parents’ sensitivity (38).

2. What is the internal linguistic structure of mothers and fathers?

Specifically, we explore the following hypotheses:

- The lack of empirical evidence in this field limits the possibility of drawing specific hypotheses. However, based on previous findings considering mothers (11), we hypothesized that mothers might reference the child’s actions and environment more often than the child’s internal state.
- On the contrary, we expected fathers to display higher levels of referents to the child’s internal states (38).

3. How is the parental language associated with the child’s clinical characteristics?

Specifically, we delineate the following hypotheses:

- We expect parents to exhibit higher levels of informative-salient speech when children have higher cognitive functioning based on the literature about typical development.
- We also expected that major amount of directive statements might be negatively associated with lower levels of the child’s development and higher levels of symptoms’ severity (47).

## 2 Materials and methods

### 2.1 Participants

*N* = 90 dyads participated in this study. In particular 45 autistic children (41 males and 4 females) were examined in interaction with their parents. Inclusion criteria for this study were (1) an autism diagnosis certified by standardized instruments (i.e., ADOS, ADI) and an independent clinician; (2) age range between 2 and 6 years and a developmental age higher than 12 months, in order to apply the clinical tests; (3) parents and children living together. Exclusion criteria of this study were (1) the absence of genetic syndromes; (2) the absence of sensory or motor impairments.

One participant presented a mental age below 12 months and was therefore excluded from the subsequent analysis. The final sample size was *n* = 44 children (41 males and 3 females) (chronological age: *M* = 41.01 months; SD = 11.96; mental age: *M* = 30.28 months; SD = 11.25) in interaction with their fathers (paternal age *M* = 41.84 years; SD = 7.02 and with their mothers (maternal age *M* = 37.37 years; SD = 5.45) (see Table 1).

**Table 1:** Descriptive statistics of the sample.

	Mean	SD	Range
Children’s chronological age (months)	41.01	11.96	22.00–68.00
Children’s mental age (months)	30.28	11.25	14.00–66.00
Mothers’ age (years)	37.37	5.45	27.00–49.00
Fathers’ age (years)	41.84	7.02	31.00–65.00
SES fathers	35.93	15.88	12.00–66.00
SES mothers	33.75	17.11	6.00–63.00

Participants were recruited at the Laboratory of Observation, Diagnosis, and Education (ODFLab – University of Trento), a clinical and research center specialized in diagnosis and intervention for individuals with neurodevelopmental disorders. The diagnosis of autism in the present work was proved through clinical judgment by independent clinicians based on the DSM-5 criteria for Autism Spectrum Disorder (ASD) and through the administration of the Autism Diagnostic Observation Schedule (ADOS-2; see measures section) (48). Further, the Griffiths Mental Development Scale Edition Revised (GMDS-ER; see measures section) (49) assessed children’s general developmental quotient and mental age. Specifically, the child’s language and communication abilities were evaluated through the specific “language and communication” scale of the GMDS-ER. Clinical observations by a psychologist and a speech therapist revealed that two children had speech-sound disorders and phonological processing disorders, and 5 children had apraxia.

Finally, the Autism Diagnostic Observation Schedule −2 (ADOS-2), which ascertains the autism diagnosis and its severity, reported a moderate ADOS Severity Score of 6.25 among children. Finally, the socio-economic status of the parents (SES mother: *M* = 33.75; SD = 17.11; SES father: *M* = 35.93; SD = 15.88) was calculated through the Four-Factor Index of Social Status (SES) (50), indicating a middle-status. Families were Italian native speakers.

Children were not involved in any rehabilitation program since the study was conducted during the first diagnostic and functional assessment, but 24 children attended daycare. In three families, children had a sibling without any neurodevelopmental disorder.

Participants were informed about the research projects through advertisements in the waiting room of the ODFLab.

Then, during the first clinical interview, the researcher explained the research objectives and the procedure in detail. Finally, families that agreed to participate in the study had to sign a written consent form. The investigation complied with the last version of the Declaration of Helsinki (World Medical Association) (51). The Ethics Committee of the University of Trento (Italy) approved all the procedures (protocol number 2020-042).

### 2.2 Procedure

During the first visits of child clinical assessment, parents were asked to play spontaneously with their children as if they were at home for ten minutes with a set of toys made by train, a toy car, a toy phone, a dinette set made of glasses, cutlery, mocha, mugs, saucers and pans, doll, ball, puzzle box, and books. The toy set was made of objects that are commonly used by all children allowing a wide variety of different play routines. The play set was made of similar toys, but the complexity and the level of sophistication were different, e.g., the book for younger children was soft with few words and larger images, whereas when children were older, the provided book had more words and phrases rather than pictures.

The interactions were video-recorded by bird’s eye cameras. In general, parents interacted with their children in different sessions with at least 7 days in between based on their availability, buffering the possible effect of the order. In the other cases (when parents came the same day), the parent’s order for the interaction was randomized.

The language was verbatim transcribed using ELAN software – Version 6.4 (52) after reaching a satisfactory level of agreement between two independent transcribers. The Penman code (see measures below) is later applied to the transcripts by two other blind independent coders after reaching very high levels of agreement (see Table 2). Finally, codes are extracted using a Python script.

**Table 2:** The Penman code and the Intraclass Correlation Coefficient (ICC) between independent coders.

Main speech categories	Intraclass Correlation Coefficient (ICC)
Affective salient speech (Expressive, generally propositional, idiomatic, or meaningless statements)	0.98
Information salient speech (Fully propositional statements about child, parent, or the environment)	0.95
Directive Statements (Types of information)	0.96
Descriptions (Types of information)	0.95
Questions (Types of information)	0.98
Child’s action (Referent of Information salient speech)	0.99
Child’s internal state (Referent of Information salient speech)	0.98
Parents (Referent of Information salient speech)	0.97
Environment (Referent of Information salient speech)	0.97
Child name (Calling the child by name or nickname in order to attract his/her attention)	0.97
Other (Non-affect or non-information speech, such as incompletely pronounced words in order to stimulate child speech. Non-codable and unintelligible speech.)	0.96

### 2.3 Measures

#### 2.3.1 Griffiths mental development scales – edition revised (GMDS – ER)

The Griffiths Mental Development Scales – Edition Revised (GMDS-ER) (49) are developmental scales used to evaluate children from 0 to 8 years old through semi-structured activities to examine different domains of children’s mental development. GMDS-ER measures the child’s mental age (MA) and developmental age in different domains, resulting in a general development quotient (GQ). The different areas investigated are five for children aged 0–2 years (e.g., locomotion, personal and social, language and communication, hand-eye coordination, and performance), and one additional subscale is added for children aged 2–8 years (e.g., practical reasoning). The scores of the scales and general quotient are standardized (*M* = 100; SD = 15). Conventionally, children are classified as without cognitive impairments if their general IQ exceeds 70 scores, whereas they are classified as children with cognitive impairments if their IQ is lower than 70.

#### 2.3.2 The autism diagnostic observation schedule – 2 (ADOS – 2)

The Autism Diagnostic Observation Schedule – 2 (ADOS – 2) (48) is a golden standard instrument for diagnosing autism and its severity. The tool has five modules according to the child’s chronological age and expressive language level. Considering our sample, we administered the Toddler module, Module 1, and Module 2.

All modules are organized into two macro-areas: Social Affect (SA), which includes communication and social interaction features, and Repetitive and Restrictive Behaviors (RRB), which includes elements related to the presence of repetitive behaviors and sensory experiences.

Moreover, social play and other unusual behaviors may be noted and evaluated.

Each module gives a total score to use in the ADOS diagnostic classification scoring system (Autism–Autism Spectrum–Non-Spectrum). This score is converted into the comparison score – used as the severity score (52) to compare different modules and classify the symptoms severity into three categories (mild, moderate, or severe). Trained clinical psychologists conducted the administration of this tool after an official ADOS course.

#### 2.3.3 Penman code

The Penman Code (12) allows the functional analysis of parental speech. This coding scheme is complete and allows a mutually exclusive assignment of subcategories since only one code can be assigned to a word or unit. Parental dialogue is codified in terms of the primary function of each vocal production (the types of purpose) and referent (to whom or to what refers).

The categories referring to the function include four main types: (1) Informative-salient speech, with the specific aim of conveying information, (2) Expressive and Affective-salient speech, to involve in affective mutual exchanges, (3) Speaking on behalf of, and (4) Naming categories. Another category is Encouraging to Speak (ET), used when the parent begins sentences or words and then stops so the child can complete them.

Informative-salient speech can also be divided into two subcategories (11, 53, 54):

1. *Type of statement:* distinguishing among questions, directive statements, and descriptive reports.
2. *Referent to the statement* refers to the child’s actions (A); refers to the child’s feelings and states (O); refers to the parent’s behaviors (P); refers to objects and other elements of the environment (E).

Even if the coding system focuses more on parental speech evaluation, specific categories refer to the child’s language. The main category includes the words clearly expressed by the child. Also, when the child is encouraged to speak by the parent and finishes the sentence, a specific code is applied to differentiate cases in which the child pronounces onomatopoeic sounds (see Table 2).

## 3 Results

### 3.1 Analytic plan

First, descriptive statistics of the data were performed. Then, data were checked for normality through the Shapiro–Wilk test and for linearity with Levene’s Test. To test differences between mothers and fathers, Independent Sample T-tests were applied in the case of parametric data, and Wilcoxon-Mann–Whitney tests were used when data were following a non-parametric distribution. Then, paired Sample *T*-tests and paired Wilcoxon-Mann–Whitney tests, were used to investigate the internal structure of speech of mothers and fathers. Socio-Economic Status (SES) was not significantly different between mothers and fathers (*W* = 74; *p* = 0.56). Further, no associations were found between SES and categories of parental speech. Therefore, the SES variable was not considered in subsequent analysis as a covariate. Finally, regression models were implemented to investigate the associations between the child’s cognitive functioning as well as the child’s symptom severity and speech characteristics of fathers and mothers. All data were analyzed using *R* software (55).

### 3.2 Similarities and differences in fathers’ and mothers’ language

First, the results (see Table 3) did not reveal any statistical difference in the total amount of words (*W* = 1188.5; *p* = 0.07) or the number of speech units (*W* = 1190.5; *p* = 0.06) that parents direct to their children. Then, to answer our first research question, we compared functional categories and subcategories of mothers and fathers. We used absolute frequencies of the main categories of parental speech for the comparison between mothers and fathers. Results revealed that mothers used more informative-salient statements than fathers (*W* = 1,259; *p* = 0.02). Further, they displayed higher levels of calling the child’s name than their counterparts (*W* = 1253.5; *p* = 0.02). As expected, results revealed no statistically significant differences in the amount of affective-salient speech (*W* = 1,153; *p* = 0.12). No other significant differences were found considering the other main functions of the language.

**Table 3:** Descriptive and inferential statistics of the main categories of paternal and maternal speech.

Main categories of speech		Fathers mean (DS)	Mothers mean (DS)	*W*	*p*
Informative	Absolute frequencies	80.02 (35.83)	104.59 (49.09)	*W* = 1,259	*p* = 0.02
	Relative frequencies	0.49 (0.12)	0.53 (0.11)		
Expressive	Absolute frequencies	47.16 (20.22)	54.16 (22.73)	*W* = 1,153	*p* = 0.12
	Relative frequencies	0.30 (0.10)	0.28 (0.07)		
Naming	Absolute frequencies	6.61 (6.47)	10.93 (9.68)	*W* = 1253.5	*p* = 0.02
	Relative frequencies	0.04 (0.04)	0.053 (0.04)		
Speaking on behalf of	Absolute frequencies	10.52 (8.81)	11.87 (8.11)	*W* = 1107.5	*p* = 0.23
	Relative frequencies	0.06 (0.04)	0.06 (0.03)		
Total amount of word	Absolute frequencies	612.91 (303.94)	740.07 (343.50)	*W* = 1188.5	*p* = 0.07
Total amount of speech units	Absolute frequencies	161.55 (59.62)	194.52 (72.10)	*W* = 1198.5	*p* = 0.06
Child language	Absolute frequencies	13.70 (16.68)	18.52 (22.99)	*W* = 1035;	*p* = 0.058

Second, we analyzed subcategories of informative-salient speech. In doing that, we considered proportional frequencies, namely the amount of a specific type of information-salient unit (e.g., descriptions, questions, and directives) with respect to the total of the main informative function. Results revealed no significant differences in any units of the informative category between fathers and mothers (see Table 4).

**Table 4:** Descriptives and inferential statistics of sub-categories of paternal and maternal speech.

Type of informative		Fathers mean (Ds)	Mothers mean (DS)		*p*
Questions	Absolute frequencies	26.68 (15.94)	34.30 (20.50)		
	Relative frequencies	0.32 (0.11)	0.32 (0.10)	*t*(83.6) = −0.049	*p* = 0.63
Directive Statements	Absolute frequencies	23.89 (14.48)	30.86 (18.63)		
	Relative frequencies	0.30 (0.12)	0.29 (0.10)	*t*(82.69) =0.34	*p* = 0.73
Reports	Absolute frequencies	29.45 (15.31)	39.43 (20.95)		
	Relative frequencies	0.38 (0.13)	0.39 (0.11)	*t*(85.21) = 0.13	*p* = 0.90
Referents of informative					
Actions	Absolute frequencies	36.11 (18.02)	47.34 (23.39)		
	Relative frequencies	0.46 (0.13)	0.46 (0.11)	*W* = 963.5	*p* = 0.97
Internal State	Absolute frequencies	5.30 (5.13)	4.45 (4.58)		
	Relative frequencies	0.07 (0.06)	0.04 (0.04)	*W* = 727	*p* = 0.04
Environment	Absolute frequencies	34.25 (21.13)	46.98 (33.55)		
	Relative frequencies	0.42 (0.15)	0.44 (0.14)	*t*(85.43) = 0.44	*p* = 0.66
Parent	Absolute Frequencies	4.32 (4.58)	5.82 (3.87)		
	Relative frequencies	0.05 (0.06)	0.06 (0.03)	*W* = 1,161	*p* = 0.11

Regarding referents of speech, proportional frequencies revealed a statistically significant difference between fathers and mothers in the number of referents directed to the child’s internal state (*W* = 727; *p* = 0.04). However, we did not find any difference in actions or environment referents between parents.

To answer our second research question, we analyzed the internal linguistic structure of both caregivers separately, and we found a similar pattern. Considering proportional frequencies of subcategories of informative speech results show a higher proportion of reports than directives (*t*(85.468) = 2.687; *p* = 0.009) and questions [*t*(82.928) = 2.3120; *p* = 0.023] for fathers. The same pattern was found in maternal language, with higher levels of reports compared to directives (*t*(85.618) = 43.881; *p* < 0.001) and questions (*t*(85.338) = 2.9764; *p* = 0.004).

Finally, when the referents were considered, the analysis of the internal structure of speech revealed that mothers directed more referents to the environment (*W* = 1936; *p* < 0.01) and actions (*W* = 0; *p* < 0.01) with respect to the referents directed to child internal states. Fathers displayed a similar pattern with higher levels of referents directed to the environment (*W* = 1920.5; *p* < 0.01) and action (*W* = 7; *p* < 0.01) with respect to the referents to the child’s internal states.

### 3.3 Child variables in association with fathers’ and mothers’ language

*To test our third research question, r*egression models were implemented to test associations between paternal and maternal language and the child’s cognitive level and symptom severity.

We fitted models with parent linguistic features as dependent variables and child characteristics as predictors. First, we found a significant model (*F* (1,42) =12.41; *p* = 0.001) indicating that the affective-salient speech of mothers is affected by the child’s cognitive functioning (*b* = −0.66; *t* = −3.52; *p* = 0.002), whereas the model was non-significant for fathers. The child’s cognitive levels influenced no other main categories for fathers or mothers.

Further, we analyzed sub-components of general intelligence. Our results revealed that maternal affective speech is influenced by the child’s linguistic abilities (*b* = −0.31; *t* = −2.49; *p* = 0.02) with a significant model (*F* (1,42) = 6.22; *p* = 0.02). However, fathers seem not to be influenced by the child linguistic abilities.

Interestingly, the child’s severity level of autistic symptoms seems not to influence the linguistic features of mothers and fathers.

## 4 Discussion

Several studies reveal that early caregiver-child interactions are relevant for child development and well-being (56, 57), and that a fundamental role is played by the parents’ language directed to their children in guaranteeing better child linguistic outcomes (58). Considering socio-communicative deficits of autistic children in this study, we focused on parental language as a specific and important aspect of the parent–child interaction.

In line with our expectations based on previous findings indicating the supportive role of mothers (59) and a style more focused on symbolic aspects during play (60), we found that mothers directed to children more informative-salient statements than fathers, revealing a linguistic style more focused on conveying knowledge.

Furthermore, the findings revealed that mothers tend to call their children more often than fathers in an attempt to recall their child’s attention when displaying non-respondent modalities due to core symptoms of autism which often flows in the tendency to reject the adult’s social initiative. These results align with previous literature investigating the maternal speech of mothers of autistic children compared to mothers of children in other clinical conditions and typical development (11). However, when fathers of autistic children are compared to fathers of children with typical development, there are no differences in their attempts to call the child’s attention (38). From this, a clear difference emerges between the two caregivers, consisting in the fact that mothers seem to show a more didactic approach aimed at conveying information compared to fathers. These results align with recent findings in which mothers displayed higher levels of unique and varied words compared to fathers, confirming a more didactic role of mothers (37).

In addition, results revealed no differences in the use of affective-salient speech displayed by the two caregivers when dealing with their children, underlying that caregivers seem to support the child through expressive verbal communication equally.

When considering specific referents of language, we found no difference in the amount of speech directed to the child’s actions and to the environment. However, we found that fathers displayed higher levels of references to the child’s internal state compared to mothers. This supports the idea that fathers could be more inclined to facilitate interaction, paying more attention to the child’s internal states and supporting child regulation (22, 23), whereas mothers are more engaged in providing cognitive scaffolding to the child. Furthermore, the fact that fathers of autistic children talk more about the internal state of their autistic children might constitute a strategy to compensate for the child’s difficulties in expressive language and self-regulation of internal states.

Despite some dissimilarities between the two caregivers, when analyzing each caregiver’s internal structure of speech, no differences emerged in the use of direct statements, questions, and reports between fathers and mothers, suggesting that both caregivers seem not to display an intrusive and directive linguistic approach while talking to their children. In fact, both parents show a similar internal language structure characterized by fewer questions and directiveness and more reports and descriptions. This may be considered an important finding that sheds light on the idea that, despite the child’s difficulties, parents support their child’s activities through verbal descriptions and verbalizations.

To conclude, two different profiles emerge from analyzing the paternal and maternal speech associated with the child’s characteristics. Considering mothers, we found that the child’s cognitive functioning influenced the affective elements of speech. In particular, mothers seem to show higher levels of expressive language toward children with lower levels of cognitive functioning. Further, when we analyzed different subcomponents of the general intelligence, results showed a tendency to use more expressive language with children with lower linguistic and communicative competencies. From our results, maternal language seems to be more influenced by the child’s cognitive features, in line with the previous findings revealing a more didactic approach of mothers compared to fathers (59). In fact, the fathers in our sample seem not to be affected by the child’s cognitive levels or symptoms severity.

In general, in line with our expectations and coherently with previous findings considering the maternal didactic role during play (60), our findings suggest that mothers use more speech addressed to provide information than fathers and seem to reinforce child linguistic and cognitive shortcomings with increased affective elements during speech. Despite this, both caregivers seem not to display an intrusive approach toward their children. In addition, both parents show a similar internal language structure characterized by fewer questions and directiveness and more reports and descriptions. This may be considered an important finding that conveys the idea that, despite the child’s difficulties, parents use descriptive language to structure the child’s activities without using intrusive verbal and non-verbal behaviors.

These findings may have important implications for understanding linguistic modalities that fathers and mothers use when interacting with their autistic children.

To conclude, fathers and mothers seem to show some similarities but also significant differences that need to be integrated into a complementary approach. The investigation of different interactive aspects in the context of autism is important in light of the recent findings that strengthen the relevance of actively involving parents in the therapeutic process with their autistic children (61–64).

A better understanding of the linguistic and communicative features displayed by parents in interaction with their autistic children may lead to implement adequate and targeted activities during the intervention. Consequently, knowing the specific characterization of the mother–child and father-child linguistic style may provide information to clinicians and practitioners to support parents in treatment optimization, focusing not only on the child’s characteristics but also considering dyadic and parents’ interactive features.

### 4.1 Limitations

Despite some interesting findings, the study poses several limitations that call for further research on this topic. First, the small sample size may prevent the generalization of the results, limiting the possibility of considering different groups according to the child’s age range. In fact, in our sample, the wide age range (22–68) of children represents a limitation that should be considered in further studies. Second, our sample was unbalanced in terms of gender according to the recent prevalence estimate between males and females.

Third, in this study, we compared mothers and fathers of autistic children without considering parents of typically developing children and/or other clinical conditions preventing us to verify if the highlighted similarities and differences between parents are specific to families of autistic children or reflect a universal tendency.

## Data availability statement

The raw data supporting the conclusions of this article will be made available by the authors, without undue reservation.

## Ethics statement

The studies involving humans were approved by The Ethics Committee of the University of Trento (Italy) approved all the procedures (protocol number 2020-042). The studies were conducted in accordance with the local legislation and institutional requirements. Written informed consent for participation in this study was provided by the participants’ legal guardians/next of kin.

## Author contributions

SP: Conceptualization, Data curation, Formal analysis, Methodology, Writing – original draft, Writing – review & editing. AB: Conceptualization, Investigation, Methodology, Writing – original draft, Writing – review & editing. SF: Supervision, Writing – review & editing. PV: Writing – review & editing.
